# 1,25-Dihydroxyvitamin D3 Ameliorates Collagen-Induced Arthritis via Suppression of Th17 Cells Through miR-124 Mediated Inhibition of IL-6 Signaling

**DOI:** 10.3389/fimmu.2019.00178

**Published:** 2019-02-07

**Authors:** Li Zhou, Julie Wang, Jingren Li, Ting Li, Yanming Chen, Rayford R. June, Song Guo Zheng

**Affiliations:** ^1^Division of Endocrinology, Guangdong Provincial Key Laboratory of Diabetology, The Third Affiliated Hospital of Sun Yat-sen University, Guangzhou, China; ^2^Department of Clinical Immunology, The Third Affiliated Hospital of Sun Yat-sen University, Guangzhou, China; ^3^Division of Rheumatology, College of Medicine, The Pennsylvania State University, Hershey, PA, United States

**Keywords:** rheumatoid arthritis, vitamin D, Th17 cells, IL-6R, miRNA-124

## Abstract

**Objectives:** To explore the molecular mechanisms in which vitamin D (VD) regulates T cells, especially Th17 cells in collagen-induced arthritis (CIA).

**Methods:** DBA1/J mice induced for CIA were intraperitoneally treated with VD. CIA clinical symptoms and inflammatory responses including Th1/Th17/Tregs percentages were determined and compared. Mouse naïve CD4^+^ T cells transduced with miR-124 inhibitor or not were polarized to Th17 cells with or without VD. Subsequently, cellular differentiation and IL-6 signaling moleculars were analyzed.

**Results:** VD treatment significantly delayed CIA onset, decreased incidence and clinical scores of arthritis, downregulated serum IgG levels and ameliorated bone erosion. VD downregulated IL-17A production in CD4^+^ T cells while increased CD4^+^Foxp3^+^Nrp-1^+^ cells both in draining lymph nodes and synovial fluid in arthritic mice. VD inhibited Th17 cells differentiation *in vivo* and *in vitro* and potentially functioning directly on T cells to restrain Th17 cells through limiting IL-6R expression and its downstream signaling including STAT3 phosphorylation, while these effects were blocked when naïve CD4^+^ T cells were transduced with miR-124 inhibitor.

**Conclusions:** VD treatment ameliorates CIA via suppression of Th17 cells and enhancement of Tregs. miR-124-mediated inhibition of IL-6 signaling, provides a novel explanation for VD's role on T cells in CIA mice or RA patients and suggests that VD may have treatment implications in rheumatoid arthritis.

## Introduction

RA is the most common inflammatory arthritis affecting up to 1% of the world population, with an even higher prevalence in women (1). It is an autoimmune disorder characterized by a chronic synovial inflammation that leads to joint destruction and functional disability in severe cases (2). While many disease modifying anti-rheumatic medications have been developed for RA, no therapy remains curative and the disease requires life-long treatment (3). Novel approaches for sustained drug free remission are sorely needed.

The mechanisms underlying RA immunopathogenesis are not fully understood; however, it is known that T cells play an essential role (4). The involvement of Th1 cells in the acute phase of the disease has been reported (5). Th17 cells, characterized by the production of IL-17, are also found in the synovium of RA patients (6), which mediates synovial inflammation through the induction of pro-inflammatory cytokines, chemokines production by macrophages, synovial fibroblasts, and chondrocytes. Th17 cells exert a direct effect on cartilage degradation and bone erosion by reducing the synthesis of collagen and proteoglycan by chondrocytes and increasing osteoclast differentiation (7). Conversely, Tregs (regulatory T cells), considered as the main regulators of the immune response by suppressing the activity of Th1, Th2, and Th17 cells (8), have also been described to be dysfunctional in RA (9).

Vitamin D has broad effects on human health that go beyond the skeletal system. Prominent non-classical effects of vitamin D are its immunomodulatory properties. Emerging clinical epidemiology evidence indicates that 30–63% of people with RA have VD deficiency, serum VD levels has been reported to negatively correlate with RA activity, and VD supplementation may ameliorate RA incidence and recurrence (10, 11). In support of this clinical epidemiological data, mice lacking functional VDR (VDR^−/−^) showed aggravated clinical signs of chronic arthritis and increased synovial inflammation (12). Furthermore, a preventive or protective effect of treatment with 1,25(OH)2D3 or with other active vitamin D metabolites on experimental arthritis (animal models of RA) has been shown (13–15). However, most previous studies have focused on incidence, severity and duration of arthritis affected by VD including detailed investigation of immunological shifts of arthritis, such as reduced frequencies of IFN-γ and/or IL-17A-expressing CD4^+^ T cells in the periphery (16, 17), and increased numbers of Treg cells (17). The underlying molecular mechanisms of VD's regulation of T cells, especially Th17 cells, in RA is unknown.

The pleiotropic cytokine IL-6 is crucial to the differentiation of Th17 cells and the balance between pathogenic Th17 and protective Treg (18). Soluble IL-6 exerts its effects by binding to a receptor complex formed by the ligand-binding IL-6Ra chain (CD126) and the signal-transducing b-subunit gp130 (CD130), which then induces homodimerization of gp130, leading to phosphorylation of tyrosine kinases of the Janus kinase (JAK) family, as well as the recruitment and activation of signal transducers and activators of transcription (STAT)-1 and STAT-3 (19). Serum and synovial fluid concentrations of IL-6 are elevated in RA (20). Inhibitors targeting the IL-6 receptor (IL-6R) improve RA disease activity, development of bone erosion, and disability socres (21, 22). Whether VD regulates IL-6 signaling in Th17 cells differentiation process remains unclear.

MicroRNAs (miRNAs) are a large class of noncoding RNAs that negatively modulate gene expression at the post-transcriptional level (23). Previous reports have shown that certain miRNAs play a role as pivotal regulators of the differentiation and function of T helper cells (24). Regarding the role of miRNAs in Th17 cells, it was reported that IL-17A production induced by TGF-β and IL-6 was reduced in Cd4CreDicer1^f/f^ cells (25). Furthermore, miR-155 and miR-326 have been shown to promote Th17 differentiation as well as severity of EAE disease (26, 27). However, the role of miRNAs in VD modulated Th17 cellular differentiation is not known.

In this study, we therefore aimed to explore the underlying molecular mechanisms by which VD regulate Th17 cells in RA, focusing on VD's effect on IL-6 signaling and the involvement of miRNAs during this process.

## Materials and Methods

### Mice

DBA1/J mice (8–10 weeks old) and wildtype C57BL/6 mice were obtained from Jackson Laboratory. C57BL/6 Foxp3^gfp^ reporter mice were generously provided by Dr.Talil Chatilla (University of Southern California, Los Angeles). DBA1/J Foxp3^gfp^ reporter mice were produced by backcrossing C57BL/6 Foxp3^gfp^ reporter mice with DBA1/J mice for 8–10 generations. All experiments using mice were performed in accordance with protocols approved by the Institutional Animal Care and Use Committee at Sun Yat-sen University.

### Induction of Arthritis

Bovine type II collagen (CII) was bought from Chondrex and emulsified with an equal volume of complete Freund's adjuvant (CFA) containing 6 mg/ml heat-denatured M.tuberculosis (Chondrex, LLC, Seattle, WA). DBA1/J mice or DBA1/J Foxp3^gfp^ reporter mice were immunized via intradermal injection at the base of the tail with 100 μl of emulsion (CII 100 μg/mouse). To determine intervention effects, mice were intraperitoneally treated with 0.5 μg/mice VD (Catalog NO.71820, Cayman Chemical) dissolved in 0.1% ethanol every other day starting 1 day before immunization for 15 days or just the same volume of 0.1% ethanol as control. This dose was selected on the basis of previous experience with mouse experiments, which was found to be effective on established CIA without producing hypercalcemia (15).

### Evaluation for Clinical Arthritis

Clinical signs of arthritis were evaluated to determine arthritis incidence every 3–4 days. Each paw was evaluated and scored individually using a 0–4 scoring system. The paw scores were summed to yield an individual mouse score, with a maximum score of 16 for each animal. Each paw score was judged as follows: 0, no signs; 1, mild swelling confined to the tarsal bones or ankle joint; 2, mild swelling extending from the ankle to the tarsal bones; 3, moderate swelling extending from the ankle to the metatarsal joints; and 4, severe swelling encompassing the ankle, foot and digits, or ankylosis of the limb.

### Histopathological Evaluation of Joints

After the animals were sacrificed on day 60, the hind limbs were collected. Following routine fixation, decalcification and paraffin embedding, tissue sections were prepared and stained with hematoxylin and eosin. All slides were evaluated by investigators blinded to the experimental conditions. The extent of synovitis, pannus formation, and bone/cartilage destruction was determined using a graded scale, as follows: grade 0, no signs of inflammation; 1, mild inflammation with hyperplasia of the synovial lining without cartilage destruction; 2 through 4, increasing degrees of inflammatory cell infiltration and cartilage/bone destruction.

### Anti-CII Antibodies ELISA

Blood were collected from each mouse on day 30 and day 60 after immunization and clotted at room temperature for 1 h followed by incubation at 4°C overnight. Sera were frozen at −80°C until analyzed. Anti-CII antibodies were measured by ELISA. Briefly, 96-well plates were coated with 200 μg/ml bovine CII overnight at 4°C. After washing four times with PBS containing 0.05% Tween-20 (PBST), the plates were blocked with 10% fetal bovine serum (Gibco) in PBS for 1 h. Samples were diluted with PBS (1:200) and added to 96 wells, incubated for 2 h at room temperature and washed once with PBST, then primed with horseradish peroxidase (HRP)-conjugated goat anti-mouse antibody. After washing seven times with PBST, the color development was primed with TMB (Sigma-Aldrich-T 0440), the reaction was stopped by 0.5 M H_2_SO_4_ and absorbance at 450 nm was measured in a microplate reader.

### Micro-Computed Tomography

Mice were anesthetized with 2% Isoflurane in oxygen. CT imaging was performed using Siemens Inveon CT scanner and Inveon Acquisition Workplace software (Siemens Medical Solutions USA Inc.). The datasets were loaded into Amira 5.2.2 and viewed using the Voltex display and the VolrenRed pseudo-color scale (Visage Imaging Inc).

### T Cell Subsets Analysis in CIA Model

Cells were isolated from draining lymph nodes of arthritic mice at day 30 and day 60 after CII immunization. For Th1 cells and Th17 cells detection, cells were stimulated *in vitro* with PMA (50 ng/ml) and ionomycin (500 ng/ml) (all from Sigma) for 5 h, with brefeldin A (10 μg/ml, biolegend) added in the last 4 h, and intracellular IL-17A, IFN-γ expression on CD4^+^ T cells was analyzed by flow cytometry. For Tregs, total cells from draining lymph nodes or synovial fluid of knee joint were stained with Foxp3 (GFP), Nrp-1 and CD4 antibodies and then analyzed by flow cytometry.

### Murine Naïve CD4^+^ T Cell Differentiation *in vivo*

CD4^+^CD62L^+^ T cells (5 × 10^5^) sorted from spleen of C57BL6 Foxp3gfp reporter mice were injected *i.p*. into C57BL6 Rag1^−/−^ mice. To determine whether VD regulates T cells differentiation *in vivo*, mice were intraperitoneally treated with 0.5 μg/mice VD dissolved in 0.1% ethanol or just the same volume of 0.1% ethanol as control every other day for 15 days starting 1 day before cells transfer. Mice developed clinical signs of colitis within 2–4 week after transfer. At day 30, mice were sacrificed and spleen, mesenteric lymph nodes (LN), lamina propria (LP) were harvested for T cells analysis as previously described (28).

### Murine Naïve CD4+ T Cell Differentiation *in vitro*

Naïve CD4^+^ CD62L^+^ T cells were purified from spleens or lymph nodes of wildtype C57BL6 mice or C57BL6 Foxp3^gfp^ reporter mice via magnetic isolation (Miltenyi Biotec, Auburn, CA). For iTregs induction *in vitro*, naïve CD4^+^ T cells were stimulated with mouse T-activator CD3/CD28 Dynabeads (cells: beads = 5:1, Invitrogen) or anti-CD3 (1 μg/ml) and anti-CD28 (1 μg/ml) in the presence of irradiated (30 cGy) syngeneic non-T cells, or immobilized anti-CD3 with anti-CD28, plus 50 U/ml rh-IL2 and 2 ng/ml rhTGF-β (all from R&D System). VD were added to cells at the beginning of cell culture with doses of 1 nM, 100 nM, or 1 uM during the *in vitro* differentiation. After 3 days or in some experiment 3/5/7 days in culture, differentiated cells were harvested and tested for Foxp3 expression. For T helper cells differentiation, naïve CD4^+^ T cells were stimulated with anti-CD3 (1 μg/ml; Biolegend) and anti-CD28 (1 μg/ml; Biolegend) in the presence of irradiated (30 cGy) syngeneic non-T cells (spleen cells washed out from nylon wool after incubated in 37°C for 40 min), or immobilized anti-CD3 with soluble anti-CD28, plus cytokines for Th1 or Th17 cell polarization differentiation as previously described (29). VD were added to cells at the beginning of cell culture with doses of 1 nM, 100 nM, 1 uM and sometimes 10 nuM during *in vitro* differentiation. After 3 days' culture, differentiated cells were re-stimulated with PMA and Ionomycin for 5 h and BFA for 4 h, IFN-γ and IL-17 expression was measured by flow cytometry. In some experiments, naïve CD4^+^ T cells were transduced with 10 nM miR-124 inhibitor (Shanghai GenePharma Co.,Ltd) for 24 h using Lipofectamine® 3000 as instruction before polarized into Th17 cells.

### Flow Cytometry Analysis

Antibodies against CD4 (GK1.5, PerCP/Cy5.5), IFN-γ (XMG1.2, APC), IL-17 (TC11-18H10.1, PE), Nrp-1 (Neuropilin-1, 3E12, PE) and CD126 (IL-6R α chain, D7715A7, APC) were from Biolegend. Synovial fluid from two knee joints of each mouse was collected and flushed out using 10 ml PBS via 1 ml insulin syringe. This method usually yields 3~10 × 10^4^ cells from arthritic mice. Results were obtained on a BD FACS Calibur flow cytometer and analyzed using FlowJo.

### RNA Isolation and Real-Time RT-PCR

RNA was isolated from differentiated T cells under Th0 or Th17 polarizing system using TRIzol reagent (Invitrogen) according to the manufacturer's protocol. cDNA synthesis was performed with TaqMan Reverse Transcription Reagents (Applied Biosystems) for mRNA or the Mir-X miRNA First-Strand Synthesis Kit (Clontech Laboratories, Inc. A Takara Bio Company) for miRNA. Quantitative PCR was performed using 2 ug total RNA and the qRT-PCR SYBR Kit (Applied Biosystems). Results were properly normalized to GAPDH or U6 snRNA levels.

### Western Blots

Purified naïve CD4^+^ cells were treated with or without VD under Th17-polarizing conditions for 48 h. In some experiments, naïve CD4^+^ T cells were transduced with 10 nM miR-124 inhibitor before polarized into Th17 cells. Whole-cell lysates were prepared in lysis buffer supplemented with protease inhibitor mix. Protein extracts were separated by 10% sodium dodecyl sulfate–polyacrylamide gel electrophoresis and stained with primary antibodies against mouse CD126/(p)STAT3 or GAPDH (Cell Signaling). Signals were detected with HRP-conjugated anti-rat or anti-rabbit IgG using the ECL system.

### Statistical Analysis

For comparison of treatment groups, we performed unpaired *t*-tests (Mann-Whitney), and one-way or two-way ANOVA (where appropriate) methods. All statistical analyses were performed using GraphPad Prism Software (version 4.01). The *p* < 0.05 is considered as statistically significant.

## Results

### CIA Progress Was Ameliorated by VD Treatment

**Figure 1:**
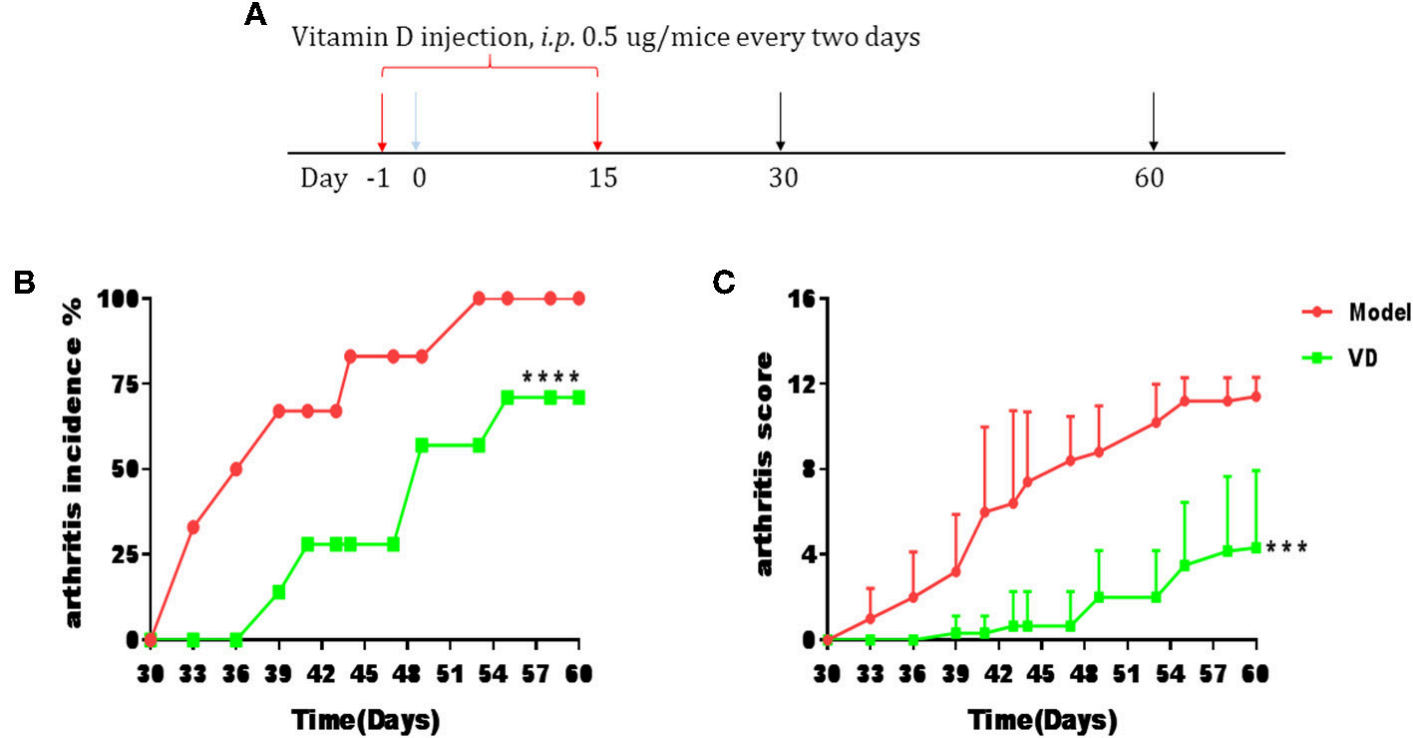
CIA progress was ameliorated by VD treatment. DBA1/J mice were immunized with collagen II (CII) emulsified with CFA to induce arthritis disease. VD dissolved in 0.1% ethanol were intraperitoneally administered to mice with a dose of 0.5 μg/mice every other day starting 1 day before immunization for 15 days and the model group were just given a same volume of 0.1% ethanol as control. **(A)** Intervention scheme for VD in CIA mice. **(B,C)** Incidence of arthritis and clinical arthritis scores. (Model, *n* = 6; VD, *n* = 7). ^***^P < 0.001, ^****^P < 0.0001 VD-treated group in comparison to CIA model group.

**Figure 2:**
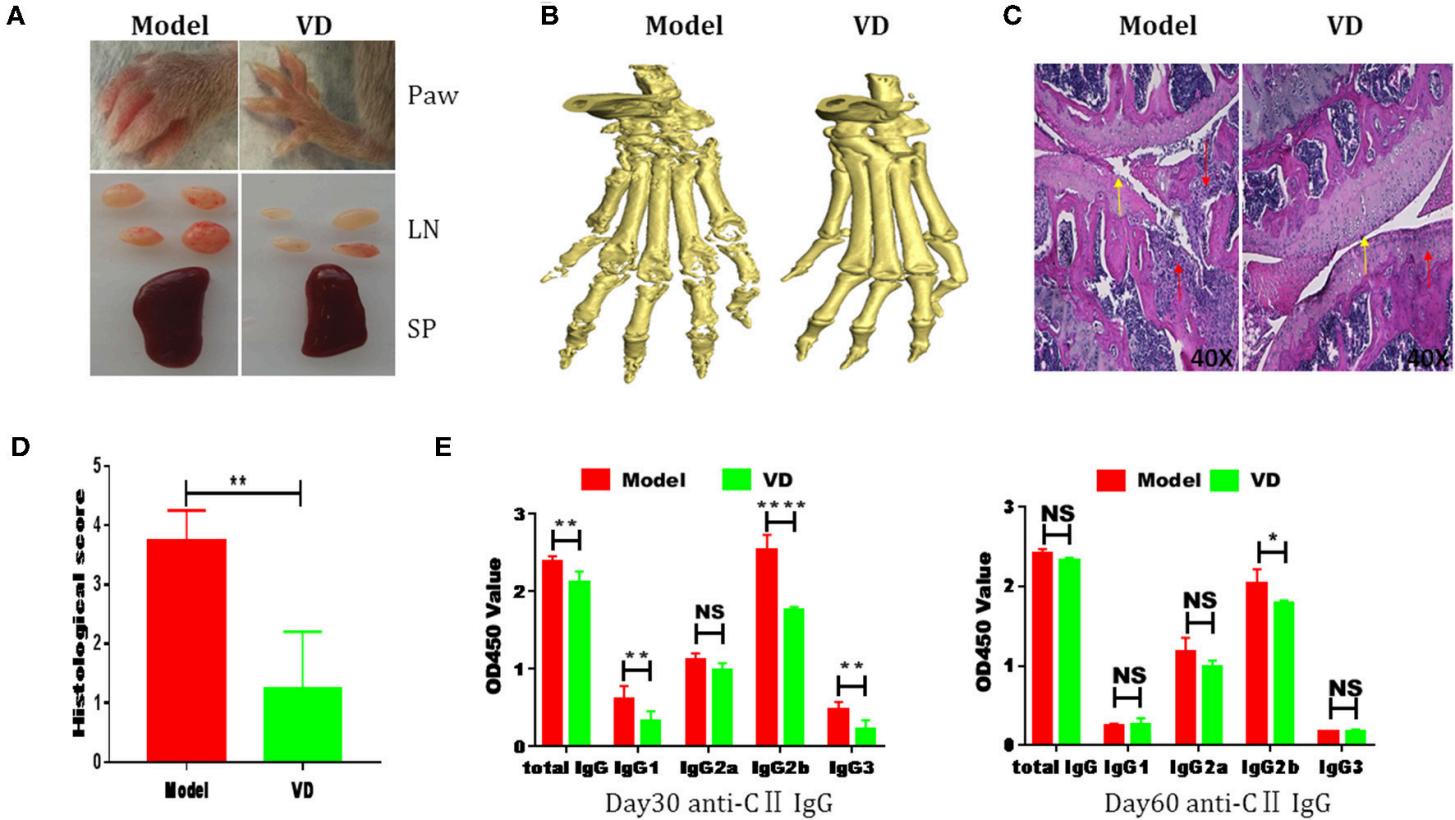
CIA severity was ameliorated by VD treatment. **(A)** Representative pictures showing the paw, lymph nodes (LN) and spleen (SP) from two groups. **(B)** Representative CT imaging of feet in CIA mice of model group and VD-treated group in 60-day after CII/CFA immunization. **(C)** Hematoxylin and eosin (HE)-stained sections and evaluation of synovitis, pannus, and erosion of knee joints in CIA mice 60 days after primary immunization. Red arrows: lymphocytes infiltration; Yellow arrows: destruction of joint cartilage. Scale bar, 40X. **(D)** The pathology scores of HE stained section were shown. Four mice were included in each group and data were combined from two independent experiments (*n* = 4). **(E)** CII-specific IgG subsets in sera harvested day 30 and day 60 after immunization were measured by ELISA (*n* = 5). ^*^P < 0.05, ^**^P < 0.01, ^****^P < 0.0001 VD-treated group in comparison to CIA model group.

The pathological features of CIA in mice are consistent with typical pathological alterations in RA patients and CIA is the most widely studied RA murine model (30). To determine the immunomodulatory role of VD in the context of autoimmune arthritis, we investigated the effect of intraperitoneal injections of VD. We observed a significant delay in CIA onset and a decrease in arthritis incidence and clinical scores following total 9 injections of VD (Figures 1A–C). CIA mice treated with vehicle developed severe joint inflammation evidenced by marked swelling and erythema of the hind paws and forepaws while VD treated mice had a remarkable decreased swelling and erythema (Figure 2A), which was consistent with micro-CT imaging results shown in Figure 2B. At day 60 after immunization VD treated mice, had nearly normal joint spacing in feet, and no evidence of bone erosion compared to the control which had the chronic destructive phase of polyarthritis in the feet and decreased spacing between the metatarsals and phalanges. Histological and quantitative analysis of whole knee joints demonstrated a significant decrease in synovitis, pannus formation and destruction of bone and cartilage in VD treated mice (Figures 2C,D). Collagen II specific antibodies are key factors in the pathogenesis of CIA (31). To better characterize the humoral immune mechanisms underlying CIA amelioration by VD treatment, we measured anti-collagen-II antibody titers of total IgG, IgG1, IgG2a, IgG2b, IgG3 in the serum of immunized mice on day 30 and day 60. Even though the levels of anti-collagen total IgG, IgG1, and IgG3 were only decreased on day 30 (Figure 2E), at both time points, we found that VD treated mice exhibited significantly lower levels of collagen-specific IgG2b Abs in comparison to model mice.

### VD Treatment Downregulated IL-17A and IFN-γ Expression While Upregulated Tregs Percentage in CIA Mice

**Figure 3:**
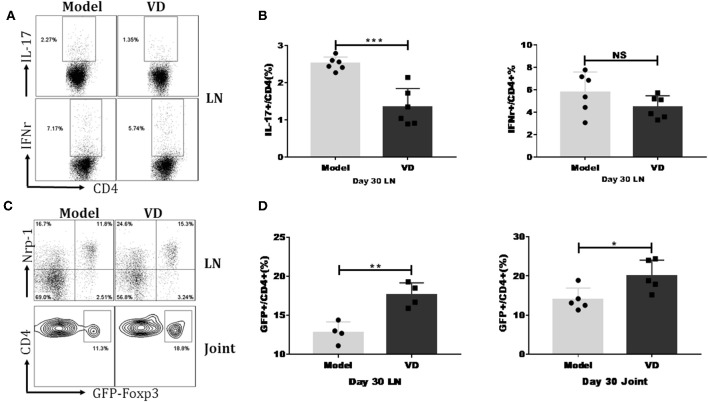
VD treatment downregulated IL-17/IFN-r expression but upregulated Tregs percentage in CIA mice. Foxp3^gfp^ reporter DBA1/J mice were immunized with CII emulsified with CFA to induce arthritis disease and VD were intraperitoneally administered with a dose of 0.5 μg/mice every other day for 15 days starting 1 day before immunization. **(A)** At day 30 after CII immunization, LN from mice inguinal and armpit were harvested and analyzed for the expression of cytokines IFN-γ and IL-17A. Representative flow data showed IL-17A and IFN-γ expression gated on CD4 positive cells of two groups. **(B)** Statistical analysis of IL-17A and IFN-γ expression on CD4^+^ T cells in the LNs of CIA mice from two groups. Data were presented as the mean ± SEM from two independent experiments (*n* = 6). ^***^P < 0.001, VD treated group vs. model group; NS, no significance. **(C,D)** Percentages of CD4^+^Foxp3^+^(GFP^+^) Tregs were tested in the LNs and knee joints on day 30 after CII immunization. Representative flow data of CD4^+^Foxp3^+^ frequency in LNs and joint synovial fluid of model and VD-treated CIA mice and Nrp-1 expression on GFP^+^ T cells of LNs, cells were gated on CD4^+^ T cells **(C)**. Statistical analysis of Tregs populations in the LNs and joint synovial fluid of mice from two groups **(D)**. Each group had four (LNs) or five (joint) mice. Data were representative of two separate experiments and mean ± SEM of each group was shown. ^*^P < 0.05, ^**^P < 0.01 vs. the model group.

We investigated the mechanisms underlying the decreased severity of CIA following administration of VD. As mentioned above, Th1/Th17 cells played essential roles in the pathology of RA/CIA, and we have previously reported that Tregs conferred significant protection against CIA (2, 32). We hypothesized that VD could shift T cells subsets to maintain immune balance. We found that VD treatment significantly reduced the percentages of CD4^+^ T cells secreting pro-inflammatory cytokines IL-17A and showed a tendency of decreased Th1 cells in the draining lymph nodes (LNs) in CIA mice on day 30 (Figures 3A,B) and day 60 (see Figure S1A).

To determine the relationship of VD with Tregs in CIA, we investigated the dynamics of Tregs in CIA mice by using Foxp3^gfp^ reporter mice on the DBA/1J strain. In line with other reports that VD treatment increases the expression of Foxp3 in atherosclerotic lesions in ApoE^−/−^ mice (33), our results revealed that VD was also able to induce Treg responses in CIA mice. In the draining LNs of CIA mice, the percentages of cells expressing Foxp3 were significantly increased at day 30 (Figures 3C,D) and day 60 (see Figure S1B) after VD treatment. We also isolated synovial fluid from two knee joints and used flow cytometry analysis to analyze T cell subsets. The Tregs population was also higher in synovial fluid from knee joints on day 30 (Figures 3C,D) although we did not see any significant difference between model and VD treated-mice on day 60 (see Figures S1C,D).

Studies have revealed that expression of Nrp-1 might distinguish thymus-derived natural Treg cells (nTreg) from induced Treg cells (iTreg) (34, 35). To identify the origin of increased Foxp3^+^ cells in VD-treated CIA mice, we co-stained cells with Foxp3 and Nrp-1 antibodies. The majority of the Tregs population in draining LNs was Nrp-1 positive in both groups (Figure 3C), suggesting that VD treatment may primarily expand endogenous nTreg cells rather than induce the generation of new iTreg cells in CIA.

### VD Treatment Inhibited T Helper Cells Differentiation While Promoted Tregs Induction *in vivo*

**Figure 4:**
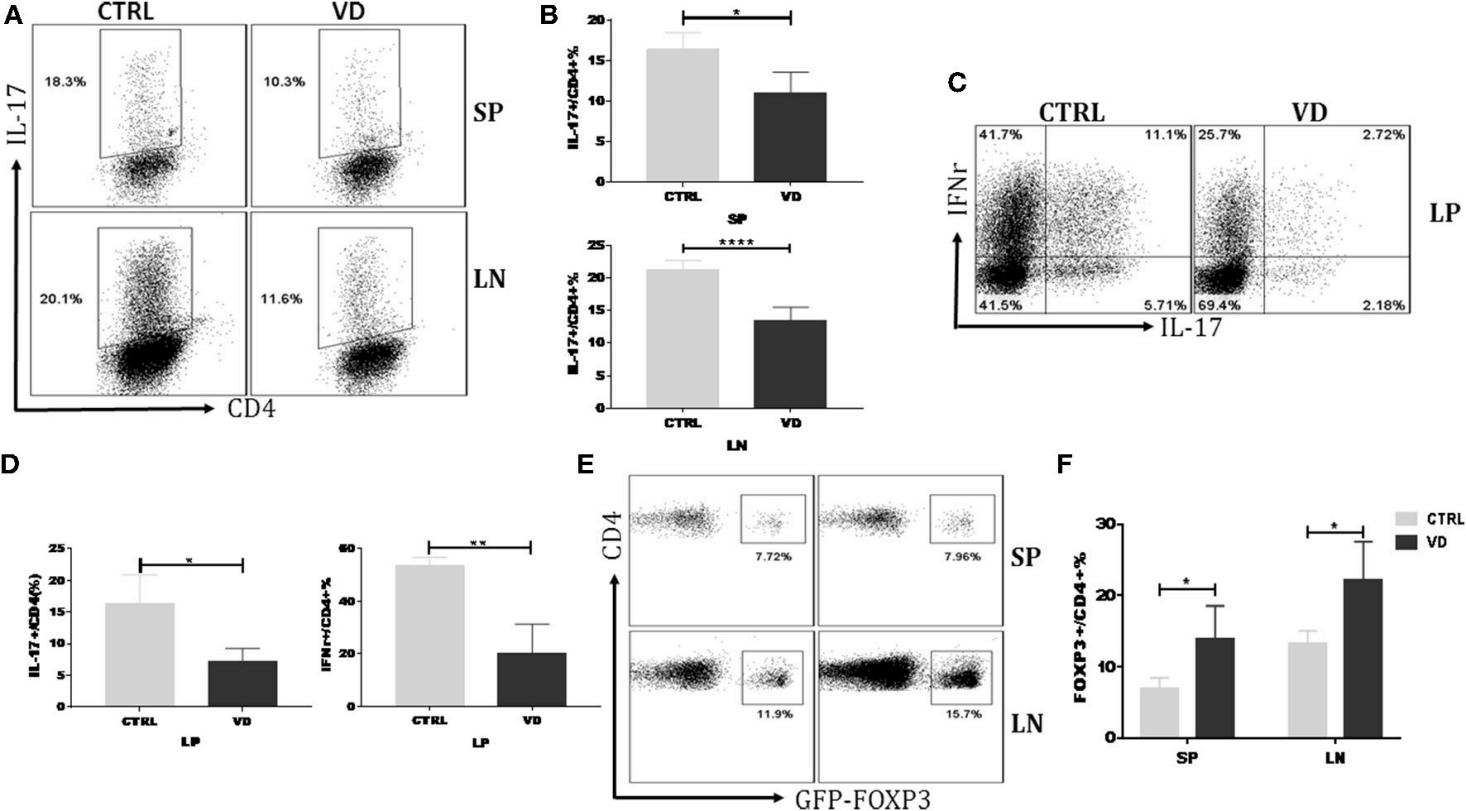
VD treatment inhibited T helper cells differentiation while promoted Tregs induction *in vivo*. MACS-sorted CD4^+^CD62L^+^ T cells (5 × 10^5^) from spleen of C57BL6 Foxp3^gfp^ reporter mice were transferred to C57BL6 Rag1^−/−^ mice through *i.p*. injection and VD was given every other day for 15 days starting 1 day before cells transfer or just the same volume of 0.1% ethanol as control. **(A,B)** Representative flow data **(A)** and statistical analysis **(B)** for the percentages of cells expressing IL-17A in the CD4^+^ population of spleen (SP) and lymph nodes (LN) at day 30 after cells transfer. Fresh cells taken from spleens and LNs of mice in the VD-treated group and control groups were stimulated with phorbol12-myristate13-acetate (PMA) and Ionomycin for 5 h and with brefeldin A (BFA) for 4 h, followed by staining for IL-17A. Cells were gated on CD4^+^ T cells. Data were presented as the mean ± SEM from two independent experiments (*n* = 4). ^*^P < 0.05, ^****^P < 0.0001, VD treated group vs. model group. **(C,D)** Colonic lamina propria mononuclear cells were isolated at day 30 and stimulated with PMA plus Ionomycin and BFA. Cells were stained for IFN-γ/IL-17A/CD4 and then were analyzed by flow cytometry (*n* = 4). ^*^P < 0.05; ^**^P < 0.01. **(E,F)** Fresh cells were taken from spleens and LNs of mice and tested for Tregs populations. Cells were gated on CD4^+^ T cells. Data are presented as the mean ± SEM from two independent experiments (*n* = 4). ^*^P < 0.05.

VD might inhibit the proliferation or differentiation of Th17 cells thus leading to a decreased Th17 cell percentage in CIA model. Figure S2 showed that VD did not significantly affect T cell proliferation even in the presence of the pro-inflammatory cytokine IL-6. To delineate further the role of VD in T cells differentiation *in vivo*, we carried out an adoptive transfer of CD4^+^CD62L^+^ cells (colitogenic cells) from C57BL/6 Foxp3^gfp^ reporter mice into Rag1^−/−^ mice to induce colitis with or without VD intervention. Naïve CD4^+^ T cells following adoptive transfer induced a high production of Th17 and Th1 cells in spleen (SP), LN and lamina propria (LP), while VD treatment significantly suppressed the generation of IL-17^+^ CD4^+^ T cells in spleen, LN (Figures 4A,B) and both IL-17 and IFN-γ expression in LP (Figures 4C,D), suggesting that VD might function through inhibiting Th17 cells differentiation to decrease Th17 population in CIA model. In this experiment, we did not observe significant change in Th1 population in SP and LN (data not shown). Although VD mainly expanded nTregs to promote an increase in Tregs in CIA model, an enhanced Foxp3 expression in spleen and lymph nodes from the *in vivo* differentiation model was also seen (Figures 4E,F), suggesting that a role for VD to induce Foxp3^−^ cells to become Foxp3^+^ T cells, i.e., iTregs, also existed.

### VD Could Function Directly on T Cells to Shape T Cell Responses

**Figure 5:**
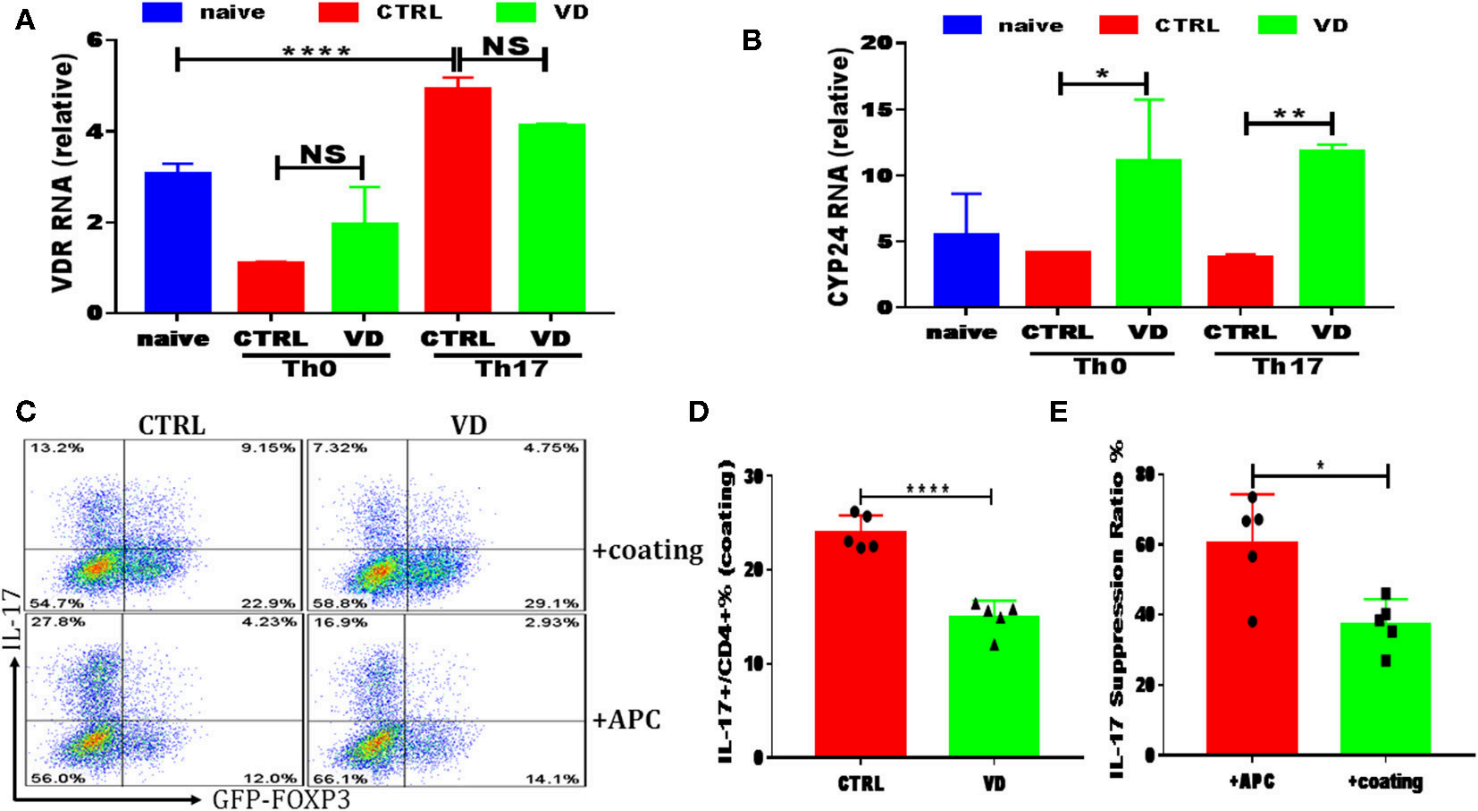
VD could also function directly on T cells to shape T cell responses. CD4^+^CD62L^+^ cells from C57BL/6J or C57BL6 Foxp3^gfp^ reporter mice were cultured in the Th0 or Th17-polarizing conditions with immobilized anti-CD3 and soluble anti-CD28 instead of APCs, in the presence or absence of 1uM VD. **(A,B)** qRT-PCR results showed VDR and CYP24 mRNA relative expression on CD4^+^ T cells stimulated in Th0 or Th17 conditions without APCs for 24 h in CTRL (without VD) and VD groups. Data are presented as the mean ± SEM. ^*^P < 0.05, ^**^P < 0.01, ^****^P < 0.0001 vs. control group. **(C–E)** naïve CD4^+^ T cells from C57BL6 Foxp3^gfp^ reporter mice were cultured in Th17-polarizing conditions with immobilized anti-CD3 and soluble anti-CD28 (+coating) or in the presence of APCs (+APCs), in the presence or absence of 1 uM VD for 3 days. Expression of IL-17A and GFP^+^ (Foxp3^+^) gated on CD4^+^ T cells were checked by flow cytometry. Representative flow data **(C)**, statistical analysis of IL-17A expression in coating system between two groups **(D)** and IL-17A suppression ratio by VD in both system were shown **(E)**. Data are presented as the mean ± SEM (experiments were repeated at least for 5 times). ^*^P < 0.05, ^****^P < 0.0001 vs. control group.

All results shown above clearly indicated that VD downregulated Th17 cells while promoting Tregs, and we subsequently used an *in vitro* system to explore the potential mechanisms. Naive CD4^+^ T cells were cultured with TGF-β and IL-6 in the presence of APCs, and the increasing concentrations of VD were added to the cultures at day 0. Consistent with *in vivo* experiments, a dose-dependent reduction in IL-17 production was observed when VD was added to culture wells (see Figures S3A,B). As for iTregs induction *in vitro* with APCs, we only saw a significant increase of Foxp3 expression in a high dose of VD (1 uM) with the presence of TGF-β (see Figures S3C,D), while no difference was revealed among groups without TGF-β even in the presence of APCs (see Figure S4). There was no significant difference in Foxp3 expression when induced with CD3/CD28 dynabeads or immobilized CD3 (see Figure S5) or IFN-γ expression in APC system or immobilized CD3 (see Figure S6). These results suggest that VD could only function through inducing tolergenetic APCs to shape T cells fate. However, we failed to observe any significant change in CD80/CD86/MHC-II expression of DC subsets in CIA mice between two groups (see Figure S7) while we demonstrated that increased VDR expression could be elicited by T cell activation of immobilized anti-CD3 plus soluble anti-CD28 under Th17 polarization conditions (Figure 5A). In addition, CYP24, an enzyme induced by VD which catalyzed synthesis of less active vitamin D metabolites as a feedback regulation of VD concentration (36), expression was enhanced by T cells treated with VD under both Th0 and Th17 conditions (Figure 5B), indicating that a direct action on T cells is likely to represent an additional or even alternative route for VD to regulate T cell responses. In line with this hypothesis, when naive CD4^+^ T cells were cultured in the Th17-polarizing condition with immobilized anti-CD3 instead of APCs, the production of Th17 cytokines was also restrained by VD treatment, although to a less degree compared with APCs system (Figures 5C–E). These results indicate that VD could directly act on CD4^+^ T cells to ameliorate CIA severity.

### VD Restrained Th17 Cells Differentiation Through miR-124 Mediated Inhibition of IL-6 Signaling

In order to investigate more precisely the direct role of VD on Th17 differentiation, we analyzed the expression of CD126, interleukin 6 receptor (IL-6R), on T cells under Th0 or Th17 polarization systems with or without VD. Purified naive CD4^+^ T cells had high CD126 expression on the surface, and CD126 gradually decreased after TCR stimulation (Th0), as previously described (37, 38). Under Th17 polarizing conditions, the addition of IL-6 significantly upregulated CD126 expression compared with Th0 conditions, while VD treatment could slow down the upregulation of CD126 on T cells (see Figures S8A,B). qPCR and western blots validated the results by flow cytometry, which showed a significant reduction of CD126 upregulation under stimulation of IL-6 and a lower expression of CD126 protein under Th17 cells differentiation system by VD compared with control, respectively (see Figures S8C–E). Thus, the downregulated IL-6R on activated CD4^+^ T cells was insufficient to activate IL-6 signaling which then resulted in inhibition of Th17 cells development.

**Figure 6:**
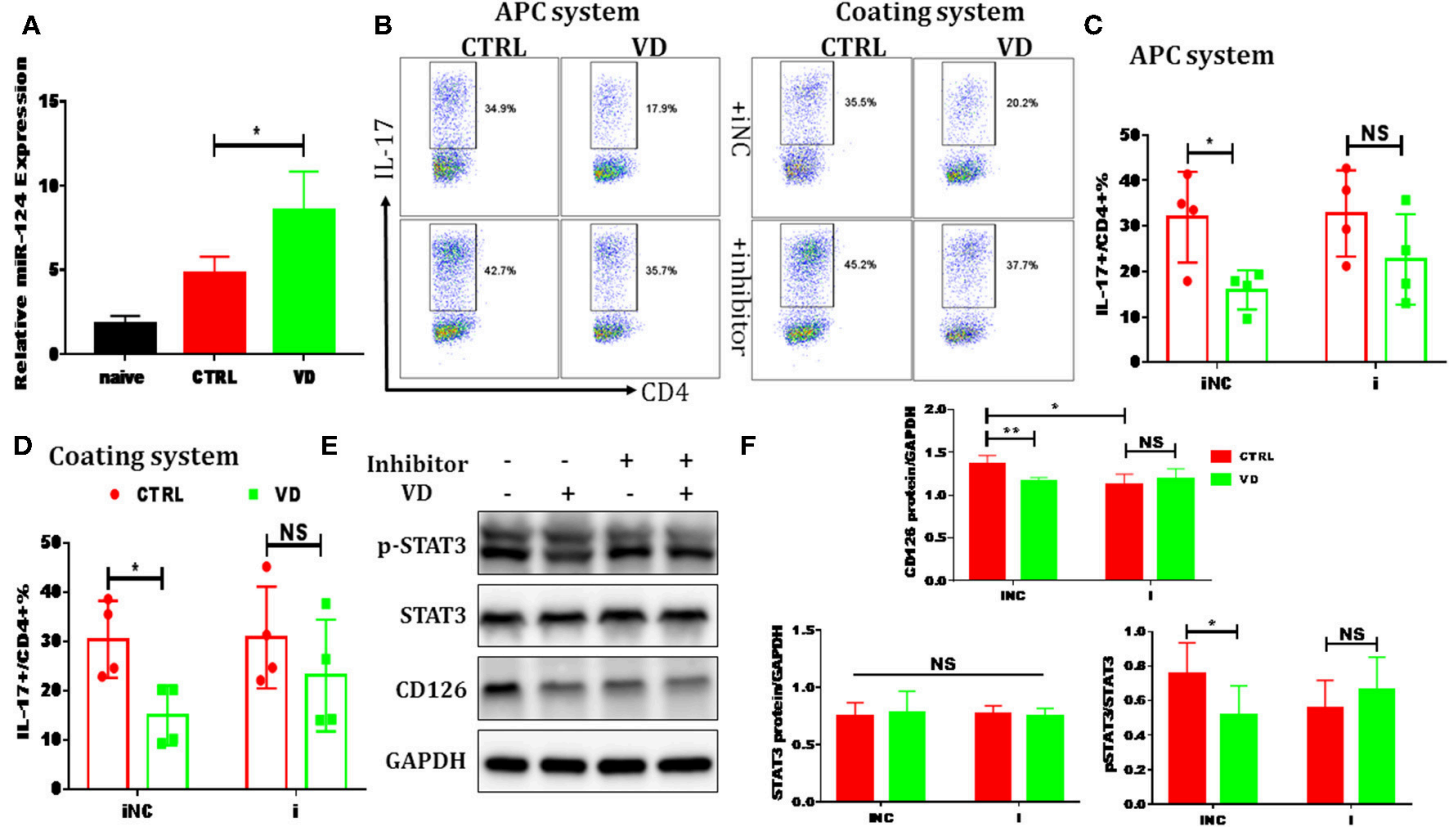
VD restrained Th17 cells differentiation through miR-124 mediated inhibition of IL-6 signaling. CD4^+^CD62L^+^ cells from C57BL/6J were cultured in the Th17-polarizing conditions with immobilized anti-CD3 and soluble anti-CD28 with or without VD. In some experiments, naïve CD4^+^ T cells were transduced with 10 nM miR-124 inhibitor for 24 h using Lipofectamine® 3000 before polarized into Th17 cells. **(A)** qRT-PCR showed that VD upregulates miR-124 expression in Th17 cells (72 h). **(B–D)** naïve CD4^+^ T cells were transduced with miR-124 inhibitor (i) or inhibitor control (iNC) before Th17-polarization using immobilized anti-CD3 or APCs system and flow cytometry was used to confirm IL-17 expression at protein level. Data are presented as the mean ± SEM (*n* = 4). **(E,F)** naïve CD4^+^ T cells were transduced with miR-124 inhibitor (+) or inhibitor control (–) before Th17-polarization using immobilized anti-CD3. CD126 expression and (p)-STAT3 activity were checked by western blots. Data are presented as the mean ± SEM. NS means no significance, ^*^P < 0.05, ^**^P < 0.01 vs. control group.

**Figure 7:**
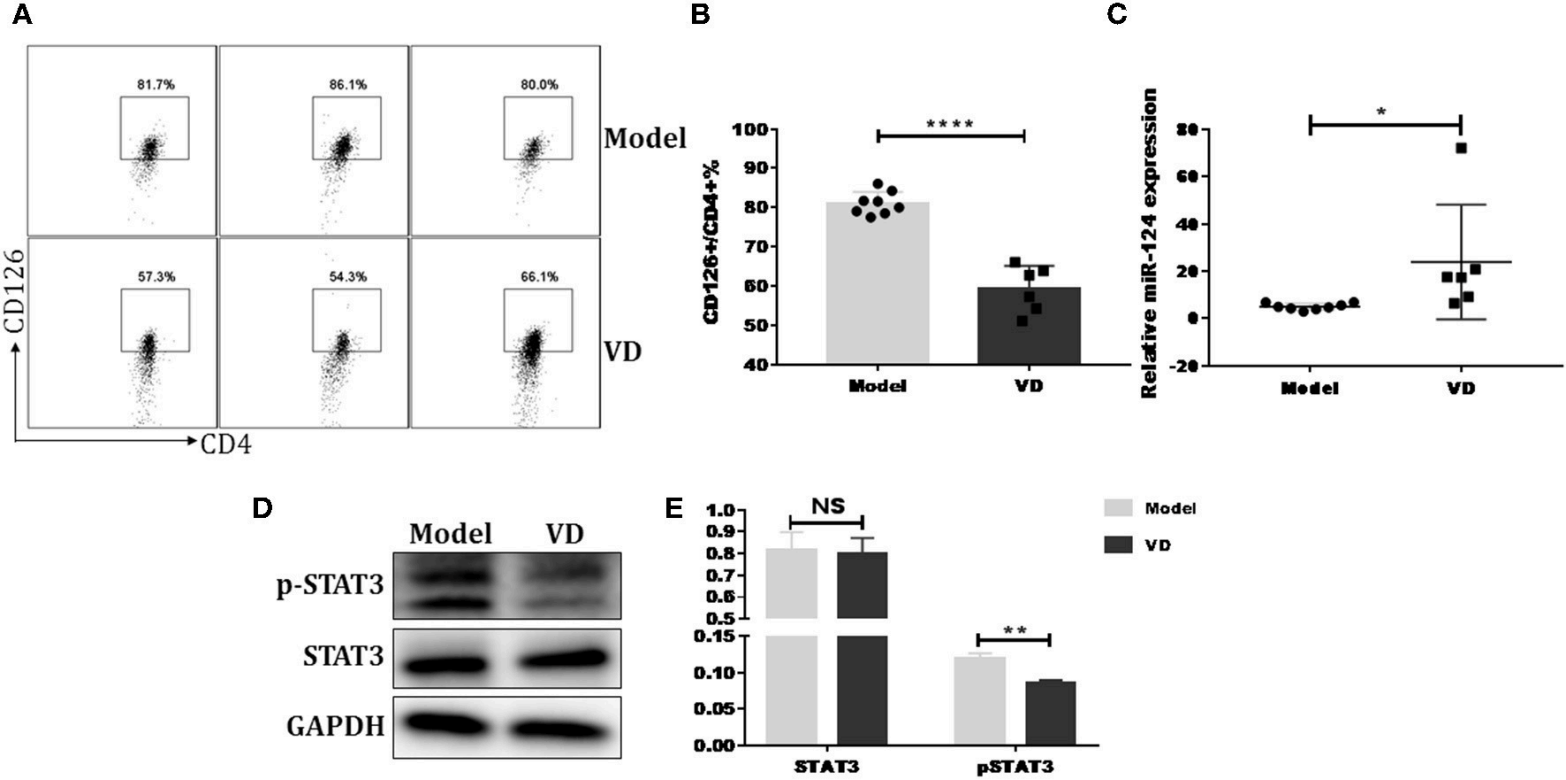
VD downregulated IL-6 signaling and increased miR-124 expression in CIA mice. DBA1/J mice were immunized with collagen II (CII) emulsified with CFA to induce autoimmune arthritis disease. VD with a dose of 0.5 μg/mouse were intraperitoneally administered to mouse every other day starting 1 day before immunization for a week and the model group were just given a same volume of 0.1% ethanol as control. **(A,B)** Representative flow data and statistical analysis of CD126 expression on spleen CD4^+^ T cells between VD and model (Model, *n* = 8; VD, *n* = 6). **(C)** Expression of miR-124 on total spleen cells was confirmed by qPCR (Model, *n* = 8; VD, *n* = 6). **(D,E)** STAT3 and (p)-STAT3 protein levels were analyzed by western blots. Data are presented as the mean ± SEM. NS means no significance, ^*^P < 0.05, ^**^P < 0.01, ^****^P < 0.0001 VD-treated group in comparison to CIA model group. Experimente were repeated with a similar result.

Previous reports have shown that certain miRNAs play a role as pivotal regulators of the differentiation and function of T helper cells and also implicated 1,25(OH)2D3 in epigenetic regulation of genes most notably as a modulator of miRNA function (39). Whether the regulatory role of VD on IL-6R and downstream IL-6 signaling during Th17 cells polarization is correlated with miRNAs is unknown. Thus, qPCR was performed to detect some miRNAs known to be closely related to T17 cell differentiation in naive CD4^+^ T cells treated with VD or not. Based on the *p* value and fold changes, miR-124 was selected for the further study (see Figure S9 and Figure 6A). Although IL-6R was not upregulated as expected when naïve CD4^+^ T cells were transduced with a miR-124 inhibitor before differentiated into Th17 cells, the downregulation of IL-6R (CD126) expression by VD treatment seen in iNC group was diminished, in accordance with similar activity of its downstream signal (pSTAT3) (Figures 6E,F) and the overall IL-17 production after transduced with miR-124 inhibitor between VD and control groups (Figures 6B–D). Finally, in accordance with *in vitro* data, we found that in CIA mice treated with VD, membrane CD126 expression on spleen CD4^+^ T cells was obviously decreased (Figures 7A,B) while miR-124 expression was increased (Figure 7C). Moreover, regardless of similar expression of STAT3, phosphorylation of IL-6 downstream signaling STAT3 was significantly decreased (Figures 7D,E). Collectively, these results clearly show that VD acts as a negative regulator for IL-6–mediated Th17 differentiation by miR-124 mediated inhibition of IL6 signaling.

## Discussion

Epidemiological studies have also reported a high prevalence of VD deficiency in people with RA, an inverse association between serum 25(OH)D concentrations and RA disease activity and a benefit of VD supplementation to RA patients or animals with experimental arthritis (10, 14, 21). In line with these reports, our study showed that VD treatment significantly delayed CIA onset, decreased the incidence, severity of arthritis, pathology scores, serum anti-CII IgG levels and ameliorated bone erosions.

An increase of Th1/Th17 cells and a defect of the numbers or function of Tregs is a key mechanisms in RA immunopathology. Several studies using VD as a supplements for RA or animal models induced for experimental arthritis demonstrated that VD functions through reducing frequencies of Th1 and Th17 cells in the periphery (16, 17) and also increasing numbers of Tregs (17) to decrease arthritis incidence and disease severity. Our study showed that the effect of VD on CIA was also dependent on down-regulating inflammatory Th17 cells while increasing CD4^+^ Foxp3^+^Nrp-1^+^ cells in draining lymph nodes and synovial fluid from knee joints in arthritic mice. Nevertheless, we only observe a tendency of diseased percentages of Th1 cells in LNs. The most probable explanation for the missing anti-inflammatory effect of VD on Th1 cells in the present study might be the time points used in this study since Th1 cells were reported to be involved primarily in the acute phase of the disease (5); it is possible that Th1 cells that were potentially decreased in the acute phase by VD treatment but had then expanded by the time points day 30 and 60.

We found that VD did not inhibit cellular proliferation even in the condition of IL-6, so a decreased population of Th17 cells in CIA mice by VD treatment might be resulted from a restrained cell differentiation. Using a naïve CD4^+^ T cells adoptive transfer colitis model, we found that VD did have a marked influence on T cells differentiation with an obvious suppression of Th1/Th17 cells and enhancement of Tregs. In the CIA mice, a similar high expression of Nrp-1 on Tregs indicated that VD treatment increased Tregs population mainly by expanding nTregs but not inducing iTregs, which was not consistent with results from adoptive transfer model. Given that we did not observe any significant differences of CD80/CD86/MHC-II expression on DCs subset between VD or vehicle treated CIA mice, a possible explanation for not inducing iTregs in CIA model might be that the ability of VD to enhance differentiation of Tregs relies on inducing tolerogenetic dendritic cells (tDCs) (40, 41), which was supported by our *in vitro* experiment showing that VD could only induce Foxp3 production in the presence of APCs and TGF-β. To our knowledge, we are the first group to explore VD's immunomodulatory function on T cells differentiation using naïve CD4^+^ T cells adoptive transfer model.

In the context of CIA, APCs might be particularly important targets for VD. However, DCs' main phenotypes had no significant change in CIA mice after VD treatment. Moreover, T lymphocytes express VDR, and upregulate its expression upon activation (42–44), suggesting that the effects of VD on CIA could be secondary to a direct effect on T lymphocytes. We conducted a series of *in vitro* experiment to analyze whether VD had direct effect on T cells and explored the underlying mechanisms by which VD shaped T cells. VD signaling transduction required its binding to VDR (45) and CYP24 expression could be induced by VD as a feedback loop in response to VD concentration (36). Our study also found that even in the absence of APCs, VDR and CYP24 expression could be increased under Th17 polarization conditions or both Th0 and Th17 conditions, respectively. Furthermore, VD had a direct effect on T cells to restrain Th17 cells differentiation, which was consistent with reports from Tang et al (46). However, we did not find any effects of VD on Tregs or Th1 cells induction in an APC-free *in vitro* system, which was contrast to other reports demonstrating that VD could also directly inhibit the production of inflammatory Th1-cytokines IFN-γ (47) and directly stimulate Tregs development (48–50). Possible explanations for this discrepancy are: (1) human cells were used to demonstrate a direct induction of Foxp3 expression by VD (48–50) and species specificity may account for the difference; (2) In some experiments, IL-10 was used as a specific marker for “Tregs,” which in fact was called Tr1 cells (type 1 regulatory T cell), instead of Foxp3 (49); (3) different culture conditions such as TCR stimulation intensity, culture time might also account for these differences.

IL-6–IL-6R–gp130-STAT3 signaling is essential for inducing the lineage-specific transcription factor retinoic acid–related orphan receptor γt that is required for Th17 cell development. Betz and Müller reported that anti-CD3/CD28 decreased the cell surface IL-6R in splenic CD4^+^ T cells (51). Our previous study also showed this phenomena and expanded the concept by demonstrating that IL-6 stimulation upregulated IL-6R expression and atRA (all-trans retinol acid) almost completely diminished IL-6R upregulation (38). In this study, we confirmed this phenotype and further characterized the functional outcome of down-modulation of this receptor in relationship to VD treatment and Th17 development by showing: (1) naive CD4^+^ T cells express high level of IL-6R on the surface, and TCR signaling downregulates the expression of this receptor protein; (2) IL-6 stimulation upregulates IL-6R expression even with TCR signaling; (3) VD treatment compromises this up-regulation in CD4^+^ T cells, which inhibits initial IL-6R signaling activity, thus leading to lower IL-17 production. Previous studies proved that: (1) miR-124 and IL-6R expression levels are inversely correlated in hepatocellular cancer cell lines; (2) miR-124 overexpression inhibits the activity of a luciferase reporter construct containing the IL-6R 3′UTR and vice versa; (3) downstream targeting of IL-6R and phosphorylation of STAT3 is induced by inhibition of miR-124 (52). Additionally, miR-124 is predicted to be a potential target of VDR (39). We found that VD treated cells and CIA mice both had a higher miR-124 expression compared with CTRL or model, which was negatively correlated with CD126 and p-STAT3 expression. In line with the results reported by Iliopoulos et al we also failed to observe the significant change of STAT3 expression. However, it was interesting that although expression of CD126 was decreased after miR-124 inhibition which was not as expected, the phosphorylation of STAT3 and production of IL-17 was similar no matter transduced with miR-124 inhibitor or not. It is well-known that sIL-6R can be generated by the shedding of membrane-bound IL-6R via limited proteolysis of the ADAM (a disintegrin and metalloproteinase) gene family members ADAM10 and ADAM17, which can also bind to IL-6 and then lead to signal transduction (trans-signaling) (53). We speculated that miR-124 inhibition might increase shedding of CD126, thus downregulated membrane CD126 but produced sIL-6R, which could also trigerring IL-6 signaling and led to STAT3 phosphorylation and IL-17 production. This hypothesis was confirmed by *in vitro* experiments (see Figure S10). Another reason for high p-STAT3 and IL-17 expression regardless of downregulated CD126 might be that miR-124 inhibition could increase the level of other molecules transducing IL-6 signaling like gp130, as gp130 is predicted to be a target of miR-124 according miRDB database, which requires further studies for verification. Depsite the unexpected decrease of CD126 expression after miR-124 inhibition, the effect on downregulation of CD126 expression or inhibition of STAT3 activity or IL-17 production by VD seen in iNC group was indeed diminished by miR-124 inhibition, which clearly demonstrated that suppression of Th17 cells by VD was associated with miR-124 mediated inhibition of IL-6 signaling.

## Conclusions

This study demonstrates that VD treatment significantly delayed CIA disease onset and decreased the severity of arthritis by downregulating inflammatory Th17 cells while increasing CD4^+^Foxp3^+^Nrp-1^+^ cells. A molecular mechanism of VD on CIA arthritis appears to be via a direct effect on T cells and/or indirectly on APCs by restraining Th17 cells differentiation via miR-124-mediated inhibition of IL-6 signaling, indicating that VD may have treatment implications in rheumatoid arthritis.

## Author Contributions

LZ: experiments conduction, data acquisition, data analysis, and wrote the manuscript; JL and TL: experiments conduction and data analysis; JW, YC: data analysis, discussion of results; RJ: manuscript review and discussion; SZ: study design, data analysis, discussion of results, and manuscript correction.

### Conflict of Interest Statement

The authors declare that the research was conducted in the absence of any commercial or financial relationships that could be construed as a potential conflict of interest.
