# Supplementary material for: 1,25-Dihydroxyvitamin D3 Ameliorates Collagen-Induced Arthritis via Suppression of Th17 Cells Through miR-124 Mediated Inhibition of IL-6 Signaling
Source: Front Immunol. 2019 Feb 7;10:178. doi: 10.3389/fimmu.2019.00178 (PMC6374300; doi:10.3389/fimmu.2019.00178)
Supplement: Supplementary file 1 [file Data_Sheet_1.PDF]

# 1,25-Dihydroxyvitamin D3 ameliorates collagen-induced arthritis via suppression of Th17 cells through miR-124-mediated inhibition of IL-6 signaling

Li Zhou, Julie Wang, Jingren Li, Ting Li, Yanming Chen, Rayford R. June and Song Guo Zheng

**Running title: VD suppresses IL-6 signaling and Th17 *via* miR-124 in CIA**

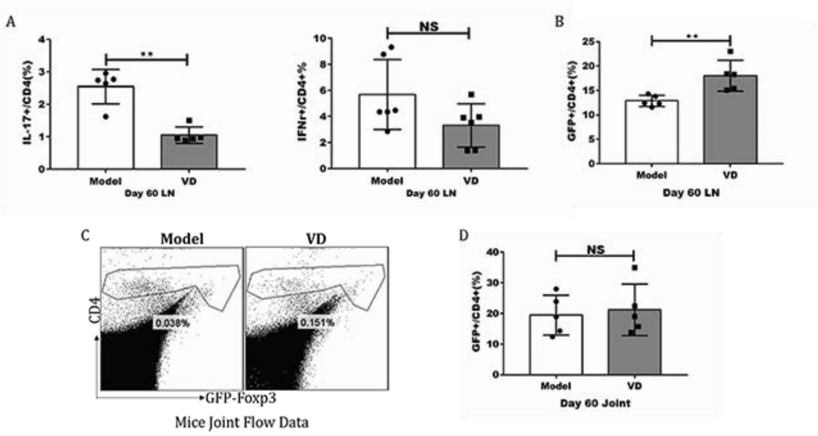


**Figure S1.**

**VD altered T cells subsets on day 60 in CIA mice.** Foxp3$^{gfp}$ reporter DBA1/J mice were immunized with CII emulsified with CFA to induce arthritis disease and VD were intraperitoneally administered. **A-C,** at day 60 after CII immunization, LN from mice inguinal and armpit were harvested and analyzed for the expression of cytokines IFN-γ, IL-17A and Tregs. **D,** Statistical analysis of Tregs populations in the joint synovial fluid of mice from two groups on day 60 (n=5).

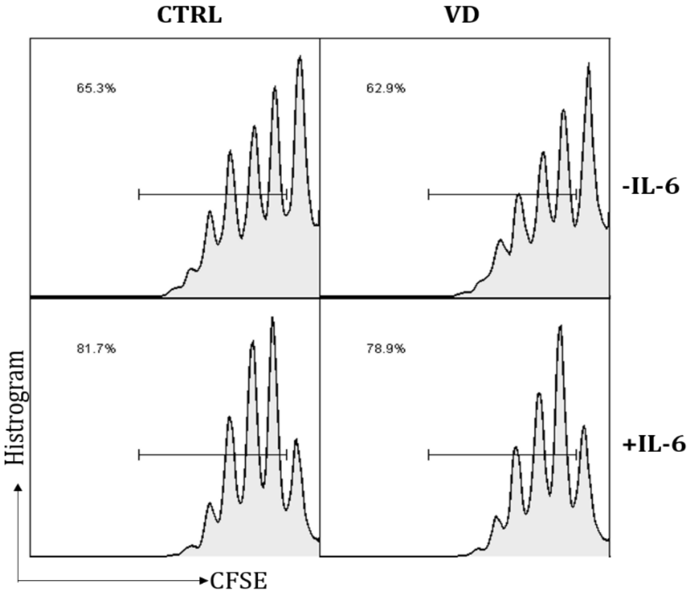


**Figure S2.**

**VD did not significantly affect T cells proliferation even in the presence of pro-**

**inflammatory cytokine IL-6.** Mouse naïve CD4$^+$ T cells were isolated and labeled with CFSE, then stimulated with anti-CD3 and irradiated syngeneic non–T cells with or without 10 ng/ml recombinant mouse IL-6 and 1uM VD. Cells were cultured for 3 days and CFSE dilution of CD4$^+$ T cells was tested by Flow Cytometry.

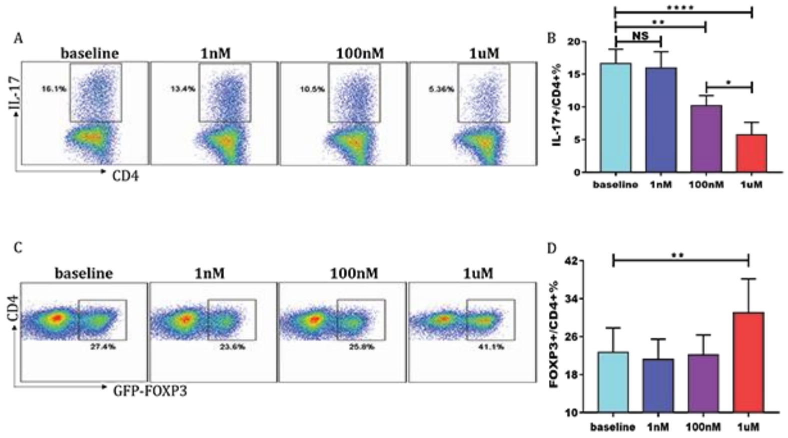


**Figure S3.**

**VD suppressed Th17 cell differentiation and reciprocally induced Treg cell differentiation in vitro in APCs system.** CD4$^+$CD62L$^+$ cells from C57BL/6J mice were polarized to Th17 cells and iTregs in the presence of APCs, with or without different concentrations of VD for 3 days. **A-B,** Expression of IL-17A gated on CD4$^+$ T cells were checked by flow cytometry. **C-D,** Expression of GFP$^+$ (Foxp3$^+$ Tregs) gated on CD4$^+$ T cells were checked by flow cytometry.

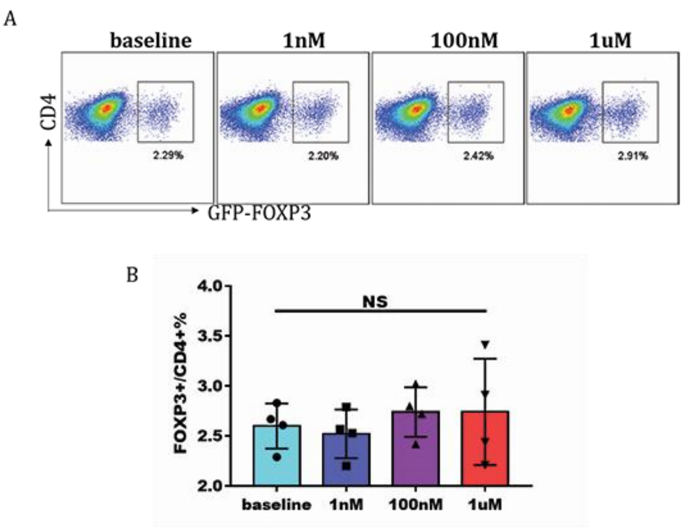


**Figure S4.**

**Foxp3 was not induced without TGF-β by VD even in the presence of APCs.** $CD4^{+}CD62L^{+}$ cells from C57BL/6 $Foxp3^{gfp}$ reporter mice were polarized to iTregs in the presence of APCs, without TGF-β for 3 days, and different concentrations of VD was added to culture systems. **A,** Representative flow data showed that Foxp3 expression were similar among groups. **B,** Statistical analysis of Foxp3 expression among groups, NS means no significance.

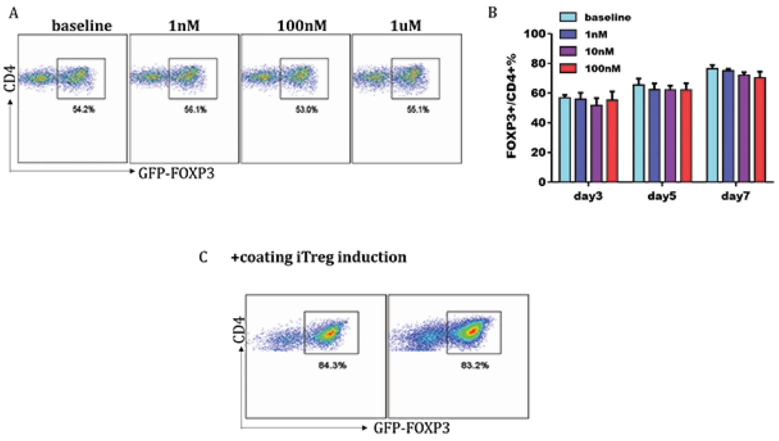


**Figure S5.**

**VD could not induce iTregs using CD3/CD28 dynabeads or immobilized CD3 system. A-B,** $CD4^{+}CD62L^{+}$ cells from C57BL6 $Foxp3^{gfp}$ reporter mice were polarized

to iTregs with CD3/CD28 dynabeads and different concentrations of VD. Cells were harvested on day 3, day 5 and day 7 for Foxp3 analysis. Representative flow data showed that Foxp3 expression were similar among groups using beads system on day 3 (**A**). Statistical analysis of Foxp3 expression among groups on day 3, day 5 and day 7 (**B**). **C,** iTregs were induced with immobilized CD3 and VD (1uM). Representative flow data showed that Foxp3 was not induced by even high concentration of VD under immobilized CD3 system.

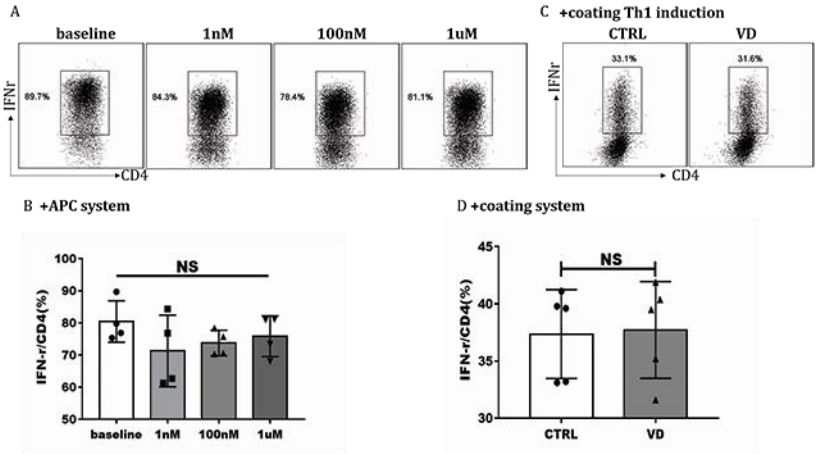


**Figure S6.**

**VD has no effect on Th1 cells induction. A-B,** $CD4^+CD62L^+$ cells were polarized to Th1 cells with APCs or immobilized CD3 (coating) and different concentrations of VD. Cells were harvested on day 3 for IFN-γ analysis. **A and B,** Representative flow data and statistical analysis of IFN-γ expression using APCs system with different concentrations of VD. **C and D,** Representative flow data and statistical analysis of IFN-γ expression using coating system with VD (1 uM).

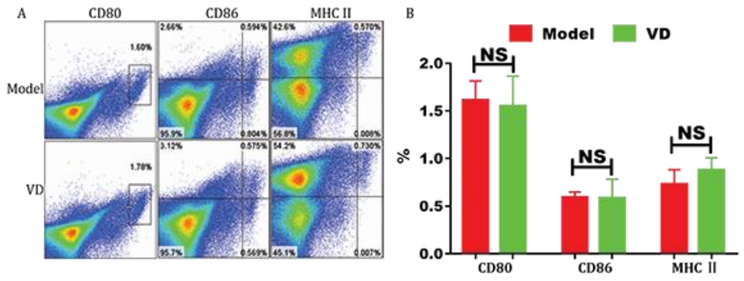


**Figure S7.**

**DCs' phenotypes in CIA mice had no significant change by VD treatment.** Foxp3$^{gfp}$ reporter DBA1/J mice were immunized with CII emulsified with CFA to induce arthritis disease and VD were intraperitoneally administered. At day 30 after CII immunization, spleens from mice were harvested and analyzed for phenotypes of DCs, including CD80, CD86 and MHC-Ⅱ. **A and B,** Representative flow data and statistical analysis of CD80 in total spleen cells and CD86 or MHC-Ⅱ expression on CD11c$^+$ cells (n=5). NS means no significance.

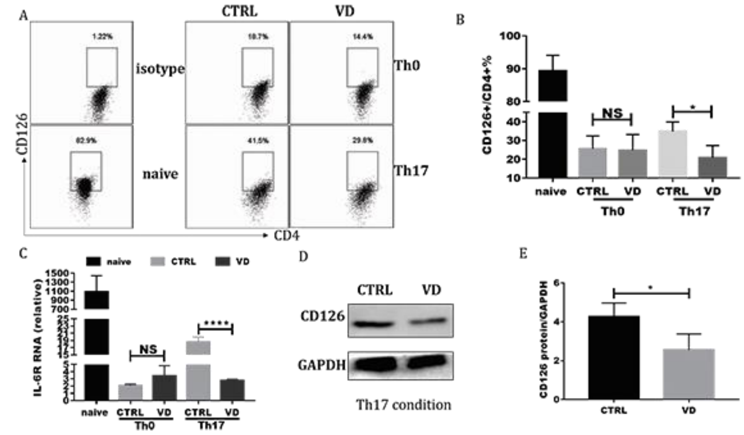


**Figure S8.**

**VD downregulated IL-6R expression to restrain Th17 cells.** CD4$^+$CD62L$^+$ cells were cultured in the Th0 or Th17-polarizing conditions with immobilized anti-CD3 and soluble anti-CD28 with or without VD (1uM). **A-B,** CD126 expression on naïve CD4$^+$

T cells and Th0/Th17 cells treated with or without VD for 24 hour was estimated by flow cytometry. **C,** IL-6R mRNA relative expression (24hour) on CD4$^+$ T cells in different situation were checked by qRT-PCR. **D,** western blots was used to confirm IL-6Rα (CD126) expression (24hour) at protein level expression. **E,** analysis for WB.

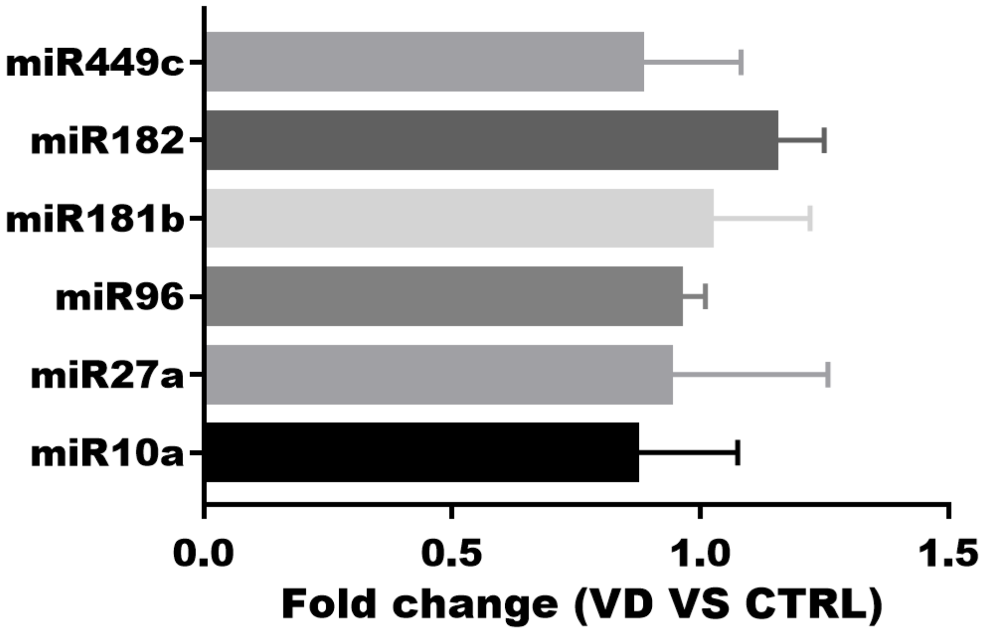


**Figure S9.**

**qRT-PCR analysis of miRNA expression profiles in naive CD4+ T cells with or without VD treatment.** CD4$^+$CD62L$^+$ cells from C57BL/6J were cultured in the Th17-polarizing conditions with immobilized anti-CD3 and soluble anti-CD28 with or without VD for 72 hours. Fold change of some miRNAs between CTRL and VD was shown.

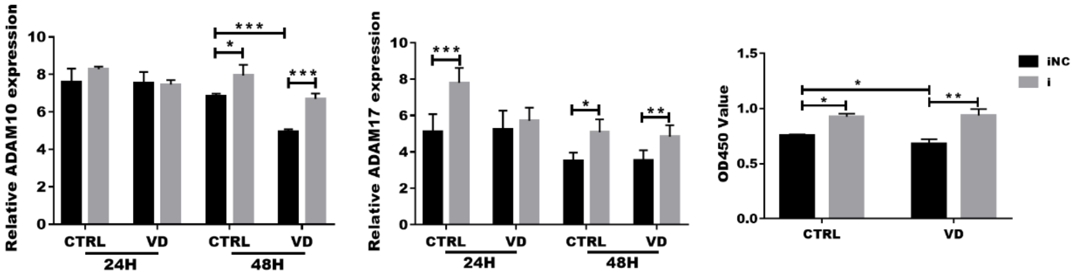


**Figure S10.**

miR-124 inhibition promoted ADAM10/ADAM17 and soluble CD126 level. $CD4^{+}CD62L^{+}$ cells transduced with miR-124 inhibitor or control for 24 hours were cultured in the Th17-polarizing conditions with immobilized anti-CD3 and soluble anti-CD28 with or without VD. ADAM10 and ADAM17 expression was measured by qRT-PCR at 24 hour or 48 hour after polarization. Supernatant at 48 hours was collected for ELISA to test soluble CD126 expression. Data are presented as the mean ± SEM. *P<0.05, **P< 0.01, ***P< 0.001.
